# An Overview of Crucial Dietary Substances and Their Modes of Action for Prevention of Neurodegenerative Diseases

**DOI:** 10.3390/cells9030576

**Published:** 2020-02-28

**Authors:** Lea Pogačnik, Ajda Ota, Nataša Poklar Ulrih

**Affiliations:** Department of Food Science, Biotechnical Faculty, University of Ljubljana, Jamnikarjeva 101, 1000 Ljubljana, Slovenia; lea.pogacnik@bf.uni-lj.si (L.P.); ajda.ota@bf.uni-lj.si (A.O.)

**Keywords:** dietary phytochemicals, endogenous substances, mitochondrial dysfunction, neurodegenerative diseases, neuroinflammation, oxidative stress, protein fibrillation

## Abstract

Neurodegenerative diseases, namely Alzheimer’s disease (AD), Parkinson’s disease (PD), amyotrophic lateral sclerosis, Huntington’s disease, and multiple sclerosis are becoming one of the main health concerns due to the increasing aging of the world’s population. These diseases often share the same biological mechanisms, including neuroinflammation, oxidative stress, and/or protein fibrillation. Recently, there have been many studies published pointing out the possibilities to reduce and postpone the clinical manifestation of these deadly diseases through lifelong consumption of some crucial dietary substances, among which phytochemicals (e.g., polyphenols) and endogenous substances (e.g., acetyl-L-carnitine, coenzyme Q10, n-3 poysaturated fatty acids) showed the most promising results. Another important issue that has been pointed out recently is the availability of these substances to the central nervous system, where they have to be present in high enough concentrations in order to exhibit their neuroprotective properties. As so, such the aim of this review is to summarize the recent findings regarding neuroprotective substances, their mechanisms of action, as well as to point out therapeutic considerations, including their bioavailability and safety for humans.

## 1. Introduction

The common feature of neurodegenerative diseases is progressive degeneration of the structure and function of the central nervous system (CNS) or the peripheral nervous system [1]. The most common neurodegenerative diseases include Alzheimer’s disease (AD), Parkinson’s disease (PD), amyotrophic lateral sclerosis, Huntington’s disease, and multiple sclerosis [2]. Several neurodegenerative diseases are characterized by abnormal protein aggregation that results from polymerization, misfolding, and subsequent aggregation of one or several peptides or proteins, as seen for AD, PD, amyotrophic lateral sclerosis, Creutzfeldt–Jakob disease, and diseases of the peripheral tissues, like familial amyloid polyneuropathy [3,4]. Progressive degeneration of neurons can lead to changes in brain function, seen as behavioral, cognitive (dementias), or motor dysfunction [5]. Treatments for neurodegenerative diseases result in great economic costs, in terms of medical and long-term care. The incidence of neurodegenerative diseases is also expected to grow, and become the second leading cause of death after cardiovascular disease [6].

Alzheimer’s disease is the most common cause of dementia, and it is a progressive and fatal neurodegenerative disease. It constitutes approximately 70% of all dementias, and its incidence increases with age, doubling every 5–10 years [7]. Patients with AD suffer from deterioration of their cognitive skills, such as memory, judgement, attention, and speech, coupled with reduced functionality for activities such as feeding, bathing, and cooking (i.e., activities of daily living). Furthermore, the associated behavioral aberrations include depression and psychiatric disturbances. The early stages of AD are often difficult to identify, and it remains nearly impossible to determine the exact point of onset [1]. Typically, several years pass between the initial onset of symptoms and death [2]. AD was first described about 100 years ago by psychiatrist Dr. Alois Alzheimer, in Bavaria [1,2]. There are several known factors for the causes of AD, from genetic origins, to advanced age, gender, and education. For example, AD is more common in women and in individuals with less formal education. In addition, a number of dietary and nutritional factors correlate with diminished cognitive skills, which include serum concentrations of folate, vitamins C, E, B_12_, and B_6_, as well as homocysteine. Some of these dietary factors represent potent antioxidants, and can reduce the oxidative stress that damages neurons and increases β-amyloid (Aβ) peptide accumulation in senile plaques; both of these consequences are believed to speed up the progression of AD [1,4]. Neurofibrillary tangles composed of hyperphosphorylated tau protein are also hallmarks of AD, as seen in post-mortem brains from these patients. Although both the amyloid and tangle pathways represent opportunities for targeted therapies against AD, the pharmaceutical industry has for many years focused only on the amyloid hypothesis [4,5].

Amyloid precursor protein (APP) is a protein that can be found throughout the body. The amyloid hypothesis says that flaws in the processing of APP in the brain lead to the production of a short fragment of APP known as Aβ. The hypothesis is based on the idea that it is the accumulation of this Aβ protein fragment in the brain that triggers the destruction of nerve cells that causes AD. Four different genes have been established to predispose to AD: APP, presenilin 1, presenilin 2, and apolipoprotein E. Presenilin 1 and 2 are integral membrane proteins and are components of *γ*-secretase. All four of these ‘AD genes’ have indicated that the excessive accumulation of cerebral Aβ is the major culprit in the pathogenesis of AD. Aβ is the main component of senile plaques and Aβ deposits in the brain [5,7].

After AD, PD is the second most common progressive neurodegenerative disease. It affects more that 1% of the human population aged 60 and over, and >5% of people by the age of 85 years [8,9]. Clinically, PD is characterized by a series of well-defined symptoms, including slowness of movement, bradykinesia, muscle rigidity, tremor, speech problems, postural instability, and decline in memory and thinking [10], which pathologically lead to death. At the cellular level, PD comes down to loss of dopaminergic (DAergic) neurons in the substantia nigra *pars compacta*, and the incidence of intracellular protein aggregates known as Lewy bodies [11,12]. PD is named after James Parkinson who described this disease in 1817, and since then there have been tremendous advances in our understanding of the etiology, pathophysiology, and genetics of PD, which have led to major breakthroughs in the development of novel and highly effective therapies [13,14]. However, there remain many unanswered questions regarding the exact mechanism of PD, and the best way to prevent the degeneration of these DAergic neurons. For PD, age is the single most consistent risk factor, and with the increasing age expectancy of the population, the prevalence of PD will inevitably rise steadily in the future [13].

It is well known that oxidative stress, neuroinflammation, mitochondrial dysfunction, and protein misfolding are the main mechanisms leading to neurodegenerative diseases. Oxidative stress is an imbalance between the production of free radical oxygen species (ROS) and the ability of the cell to detoxify the free radicals. While the exact mechanisms of PD pathogenesis still remains known, it is believed that aggregation of the presynaptic protein α-synuclein has a critical role in its etiology [1,2,3]. The exact function of α-synuclein is unknown. Based on the fact that a significant fraction of α-synuclein is localized within membrane fractions, and especially in synaptic vesicles associated with vesicular transport processes, it is likely that α-synuclein has a role in vesicular trafficking [4,5,7,15]. Under physiological conditions in vitro, wild-type α-synuclein is an intrinsically disordered protein that consists of 140-amino-acid residues. The polypeptide chain of α-synuclein includes a highly conserved N-terminal domain (residues 1-95) with seven 11-amino-acid residues that are long imperfect repeats with a consensus sequence of KTKEGV, as a lipid-binding motif. Recently, nuclear magnetic resonance studies showed that α-synuclein can adopt an ensemble of conformations that are stabilized by long-range interactions [16]. In particular, a long-range intramolecular interaction between the C-terminal region (residues 120–140) and the central part of α-synuclein (residues 30–100) has been reported [16]. It is believed that this interaction, which can be electrostatic or hydrophobic (or both), inhibits fibrillation of α-synuclein.

Some epidemiological studies showed that diets that are rich in fruit and vegetables can reduce the incidence of non-infectious diseases, such as diabetes, cancers, cardiovascular diseases, and stroke. These is some evidence that the observed positive health effects can be at least partially assigned to phenolic secondary metabolites. Recently, various polyphenols in medicinal herbs and plants have been attracting lots of attention for use in human health [17,18]. The most abundant natural polyphenols from fruit, vegetables, and tea are flavonoids. It was shown that they can block many proinflammatory proteins and function as natural inhibitors of inflammation [19]. A systematical review by Moosavi et al. [20] also discussed the effects of polyphenols on viability of neurons, their growth, proliferation, and differentiation. Moreover, the signaling pathways involved in these neurotrophic actions are also reviewed. Here, we focus on the neuroprotective properties of polyphenols against the main pathophysiological properties of most neurodegenerative diseases, namely oxidative stress, neuroinflammation, protein fibrillation, and mitochondrial dysfunction.

This review gathers together several of the most promising phenolic compounds currently under study, with a view towards treatment and prevention of AD and PD. Perhaps the most important aspect for the consideration of polyphenols as a potential treatment avenue for neurodegenerative diseases is their ability to enter the CNS. In this respect, the important information is also their ability to accumulate in brain tissue at high enough concentrations and in a biologically active form. The main difficulties that polyphenols encounter during transportation to the CNS are the blood-brain barrier (BBB) and multidrug-resistance-associated proteins. Until recently, the knowledge of ability of polyphenols to cross the BBB was relatively weak. Nevertheless, it is now proven that a various polyphenols can indeed cross the BBB [21,22].

## 2. Neuroprotective Substances

### 2.1. Dietary Phytochemicals

**Figure 1:**
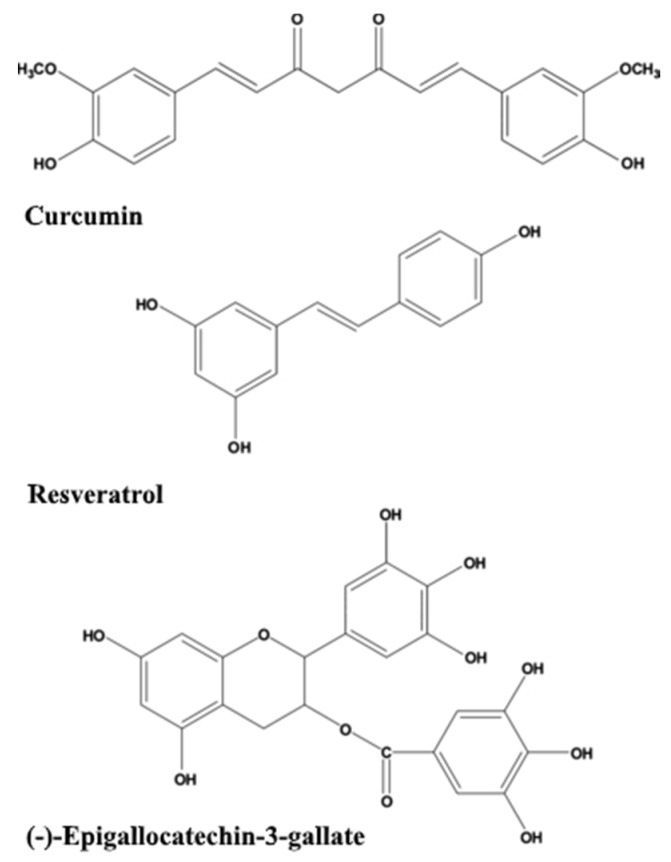
The structures of curcumin, resveratrol, and (−)-epigallocatechin-3-gallate.

Dietary phytochemicals that have antioxidant activities as well as anti-amyloidogenic properties are being investigated for their potential beneficial effects, such as curcumin, resveratrol, green tea catechins such as (−)-epigallocatechin-3-gallate (EGCG) (Figure 1), sylimarin, ginkgolides, and flavonoids from *G. biloba* [23,24,25], as detailed in the following sections. Several recent reviews [26,27,28] focus on mechanisms of their action as neuroprotective substances. An interesting study showed that total urinary polyphenols were associated with lower risk of substantial cognitive decline and cognitive decline in an older population [29].

#### 2.1.1. Curcumin

Curcumin (diferuloylmethane) is a phytopolyphenol pigment that can be isolated from *Curcuma longa*, a perennial flowering plant of the ginger family, Zingiberaceae. *C. longa* and its derived spice are commonly known as turmeric, and it has been used for centuries in Indian and Chinese medicine [30]. Curcumin has several favorable features as a neuroprotective drug, which include antioxidant, anti-inflammatory, and anti-protein-aggregation activities. As a result, curcumin shows a high ability to prevent several neurological conditions [31]. The Indo-United States (US) Cross-National Dementia Epidemiology Study demonstrated that the Indian population with a diet rich in curcumin has a reduced prevalence of AD compared to that in the US [32], however the direct association between the two has not yet been proven. Recently, it was shown that curcumin represents a promising anti-inflammatory, neuroprotective, and anti-amyloid agent and thus can be used for treatment of several neurodegenerative diseases. With its pleotropic actions in the CNS (e.g., its preferential binding to amyloid protein), curcumin is being proposed as a promising substance for age-related brain diseases [33]. In vitro studies have demonstrated anti-amyloidogenic properties of curcumin and its analog rosmarinic acid, with dose-dependent inhibiting of the formation of Aβ fibrils, destabilization of preformed Aβ fibrils, and regeneration of Aβ monomers [34]. Curcumin prevents oxidative damage in the brain through its scavenging of nitric oxide (NO)-based radicals and hydroxyl radicals, as well as through its binding to redox-active metals and its prevention of inflammation by inhibition of transcription of the inflammatory cytokines, and of inducible NO synthase (iNOS) and cyclooxygenase 2 (COX-2) [31,34]. The anti-AD effects of curcumin have been demonstrated in several murine and rat models of AD, and it thus warrants further research and clinical studies of its use as a dietary supplement for prevention and treatment of AD [35,36,37]. However, while curcumin appears to have potential clinical benefits, its relatively low bioavailability has already been highlighted by several authors [38,39,40,41].

#### 2.1.2. Resveratrol

Resveratrol (3, 5, 4′-trihydroxystilbene) is a phytoalexin, naturally produced by plants as part of their defense mechanism, which is activated in the case of infection and/or injury caused by bacteria, fungi, UV irradiation, and other stresses [42]. Resveratrol has two isomeric forms, and it is the most abundantly present in grape skins, berries, Japanese knotweed, red wine, rhubarb roots, and peanuts [43,44]. In vitro and in vivo studies have demonstrated that resveratrol readily crosses the BBB and can have positive effects in neurodegenerative diseases such as PD, AD, cerebral ischemia, prion disease, epilepsy, Huntington’s disease, and amyotrophic lateral sclerosis [45,46,47]. Resveratrol has demonstrated anti-amyloidogenic activity through reduction of the levels of secreted and intracellular Aβ in vitro [48]. Resveratrol has a neuroprotective action against oxidative stress through its scavenging of free radicals and its up-regulation of cellular antioxidants [49,50]. The neuroprotective effects of *trans*-resveratrol have also been seen in animal models, where prevention of cognitive impairment and spatial memory deficits have been reported [51,52]. One of the main limitations of the use of resveratrol has proven to be its low oral bioavailability, which is the result of its extensive metabolism and rapid excretion [53]. However, the large amount of the data that describe the neuroprotective properties of resveratrol support an interest in this polyphenolic compound as a viable therapeutic approach for the treatment of severe neurodegenerative diseases [54,55,56].

#### 2.1.3. Green Tea Catechins

Tea is one of the highest quantity beverages consumed throughout the world. An increased interest in the health properties of medicinal plants has led to a significant increase in studies of the health benefits and medicinal properties of tea [57]. The beneficial effects of green tea (*Camellia sinensis*) have been attributed to the catechins, which are phenolic compounds that show powerful anti-oxidant, iron-chelating, and anti-inflammatory activities [58]. The major catechins in green tea include: (–)-epicatechin, (–)-epigallocatechin, (–)-epicatechin-3-gallate, and (−)-epigallocatechin-3-gallate (EGCG) [59].

**Figure 2:**
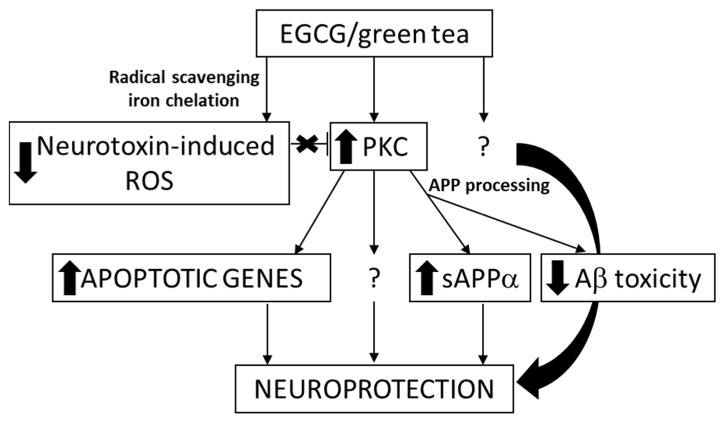
The suggested targets involved in the neuroprotective actions of EGCG [68].

EGCG is also the major catechin that can be extracted from the polyphenolic fraction of green tea, where it accounts for 50% to 80% of the catechin content [60]. Several positive biological actions of this polyphenol in addition to its antioxidant properties, appear through specific mechanisms of action that include its interaction with proteins and specific receptors from the cell-surface membrane, and its regulation of cell signaling pathways and transcription, mitochondrial function [19,61], DNA methylation, and autophagy [62]. It was shown by in vivo studies in mice that EGCG reduces Aβ generation [63], modulates amyloid precursor cleavage, and reduce cerebral amyloidosis, and that it inhibits Aβ-induced cognitive dysfunction through modification of secretase activity [64,65]. The same polyphenol facilitates T-cell nuclear factor-κB inhibition and provides neuroprotection in autoimmune encephalomyelitis, an animal model of multiple sclerosis [66]. The potential targets involved in neuroprotection caused by EGCG are presented in Figure 2. The epidemiological studies on the association between tea consumption and the reduced risk of AD are reviewed and the anti-amyloid effects of related bioactivities in tea are summarized by Polito et al. [67].

For EGCG, there is also epidemiological evidence that supports a link between the intake of green or black tea and reduced risk of developing PD [58]. A randomized, double-blind, placebo-controlled epidemiological study was performed to characterize the effects of green tea polyphenols, including EGCG in PD. The research was financed by The Michael J. Fox Foundation and conducted by the Chinese Parkinson Study Group. The aim was to evaluate the efficacy and safety of green tea catechins in patients with de novo PD. The study involved 400 untreated people with PD and showed significant improvement after 6 months for each dose group [69]. It is also important to stress that no adverse health effects were observed in any of the groups that consumed green-tea polyphenols. However, after 12 months these improvements were no longer significant compared to for the placebo-treated group. Therefore, it was concluded that green-tea polyphenols have no noticeable disease-modifying outcomes, even though the symptomatic relief was observed in early de novo PD. As such, the amount and frequency of administration of polyphenols, including EGCG, is one of the main issues that needs to be resolved before applying these substances in human therapies [70].

#### 2.1.4. Sylimarin

Silymarin is a mixture of polyphenolic compounds extracted from the dried fruit of the milk thistle, *Silybum marianum*, and it has been commonly used since ancient times for its hepatoprotective activity [71,72]. Among the phytochemicals, silymarin is one of the most extensively used flavonoids, due to its potential therapeutic characteristics [73]. It has been used at treatment of various pathological conditions of the lungs, prostate, skin, CNS, pancreas, and liver. Silymarin comprises of various flavonolignans (i.e., silybin, silychristin, isosilybin), as well as low quantities of flavonoids (e.g., taxifolin), other polyphenolic compounds with strong antioxidant capacities, and fatty acids. Various models of neurological disorders have been used to study the neuroprotective effects of silymarin, including those for AD, PD, and cerebral ischemia. The bioavailability of silymarin is low, and due to its poor aqueous solubility, only 23% to 47% of it has been shown to reach the systemic circulation after oral administration [9]. It was shown that silymarin reduces the activation of microglial cells and also the production of inflammatory mediators, namely tumor necroses factor-alpha (TNF-α) and NO, with reduced damage to DAergic neurons as a result [74]. As reported previously, silymarin maintains striatal DA levels through decreased apoptosis in the substantia nigra, and the consequent preservation of the DAergic neurons. Several studies have connected these findings to the antioxidant capacity and anti-inflammatory potential of silymarin [75]. Some studies have reported that silymarin also reduces the levels of α-synuclein and increases the levels of DA [9]. A comprehensive review of the recent literature exploring the effects of silymarin administration on the progression of PD was published recently. In the review, the authors are focused on the chemical basis of the pharmacology of silymarin in the treatment of PD, as well as its mechanisms and possible therapeutic/clinical status while addressing the bioavailability limitation [9].

#### 2.1.5. Gingolides from *Ginkgo biloba* L.

**Figure 3:**
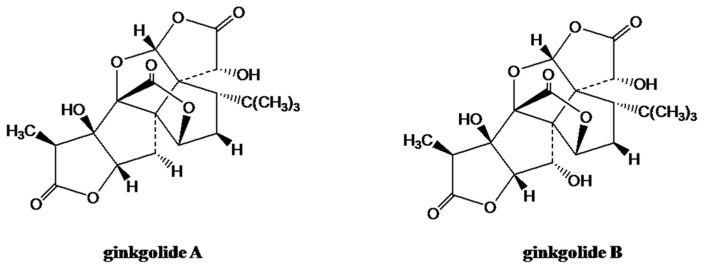
Ginkgolides A and B (adapted from Reference [81]).

*Ginkgo biloba* L. is a dioecious tree that belongs to the Ginkgoceae family, and it is the oldest living species. Thus *G. biloba* is also called a ‘living fossil’, and it is traditionally used in Chinese herbal medicines [76]. Clinical studies have revealed that ginkgo extracts have therapeutic activities for a several different diseases, including age-related dementias, failing memory, eye ailments, vascular insufficiencies, congestive symptoms of premenstrual syndrome, and oxidative stress [77,78]. A standardized *G. biloba* extract (EGb 761) has been used as a natural treatment for cardiovascular and neurodegenerative diseases [79]. It consists primarily of the trilactone terpene ginkgolides (Figure 3) and flavonoids [80].

A *G. biloba* leaf extract has been shown to protect against Aβ-induced neurotoxicity through inhibition of the formation of Aβ-derived diffusible neurotoxic soluble ligands, which are reported to be involved in the pathogenesis of AD [82]. Several clinical studies have reported that this *G. biloba* leaf extract can improve cognitive and noncognitive symptoms of dementia [83,84,85,86]. The antioxidant properties and the enhanced activities of antioxidant enzymes are believed to contribute to the therapeutic actions of this *G. biloba* leaf extract [76]. Preclinical studies performed both in vitro and in vivo involving AD and PD show that G. biloba leaf extract (EGb761) has a neuroprotective/antioxidant effect [87]. The use of EGb761 as an antioxidant agent in clinical trials has not been explored directly, but indirect information supports its use to both neurodegenerative diseases. Further clinical trials are needed to explore the potential clinical use of EGb761 in the treatment of patients suffering from these health conditions.

### 2.2. Endogenous Substances

#### 2.2.1. Acetyl-l-Carnitine

Acetyl-l-carnitine has essential roles in intermediary metabolism, where it acts as a donor of acetyl groups and is involved in the transport of fatty acids across the mitochondrial membrane during β-oxidation [88]. Dietary supplementation of acetyl-L-carnitine has been shown to have neuroprotective, neurotrophic, antidepressive, and analgesic effects in painful neuropathies [66]. The main mechanism of action of acetyl-L-carnitine is improved mitochondrial respiration, which allows the neurons to generate the necessary adenosine triphosphate (ATP) to maintain the normal membrane potential [89]. In vitro studies have demonstrated dose-dependent neuroprotective activities of acetyl-L-carnitine against Aβ-induced neurotoxicity, protein oxidation, lipid peroxidation, and apoptosis [90,91]. Neuroprotective and neurotrophic activities of acetyl-L-carnitine have also been demonstrated in primary cultures of rat embryo motor neurons, with improved activity seen [92]. Double-blind controlled studies have also reported beneficial effects of acetyl-L-carnitine in major depressive disorders and AD, two major psychiatric disorders at high prevalence in the geriatric population [93]. A meta-analysis carried out by Montgomery et al. concluded that acetyl-l-carnitine can improve the conditions of patients with mild cognitive impairment and early AD [94]. The cumulative data gathered from in vitro and in vivo studies thus show that acetyl-L-carnitine might prove to be an excellent drug for the treatment of AD and of other neurodegenerative diseases, especially as it is an endogenous substance. A comprehensive review of acetyl-L-carnitine and its induced neuroprotective and neurotrophic effects and their role in the brain has been published recently [88].

#### 2.2.2. Coenzyme Q10

Coenzyme Q10 (ubidecarenone) has a central role as an electron carrier in the mitochondrial respiratory chain, and it acts as a potent antioxidant both in the mitochondria and in other cellular membranes. Coenzyme Q10 is therefore an attractive endogenous compound for use in the treatment of neurodegenerative diseases where there is evidence of dysfunction of complexes I and/or II, or of excessive oxidative stress [95]. These processes have been implicated in the pathogenesis of several neurodegenerative diseases, including PD, Huntington’s disease, and Friedreich’s ataxia [96]. Coenzyme Q10 has been shown to reduce damage in animal models of a number of neurodegenerative diseases [97]. Recent data have indicated that coenzyme Q10 affects the expression of genes involved in cell signaling, metabolism, and transport, and so some of the effects of its supplementation might be due to these properties [98]. Clinical trials have suggested that coenzyme Q10 supplementation can decrease inflammatory markers in patients with multiple sclerosis, and it appears to slow the progressive deterioration of function in PD [99,100]. As coenzyme Q10 is well tolerated and new formulations have been developed that increase its bioavailability, it might have beneficial effects in the treatment of neurodegenerative diseases. Lipophilic antioxidants, such as lipophilic vitamins (vitamin A, vitamin E), carotenoids, coenzyme Q10, and n-3 polyunsaturated fatty acids (e.g., docosahexaenoic acid and eicosapentaenoic acid), have received increasing attention as part of therapeutic and preventive intervention for neurodegenerative diseases. Recently, a review covering this subject has been published [101]. Through findings in cell and animal models, as well as biomarker studies, the relationship between lipophilic antioxidants and neurodegeneration has been pointed out.

#### 2.2.3. n-3 Polyunsaturated Fatty Acids

**Figure 4:**
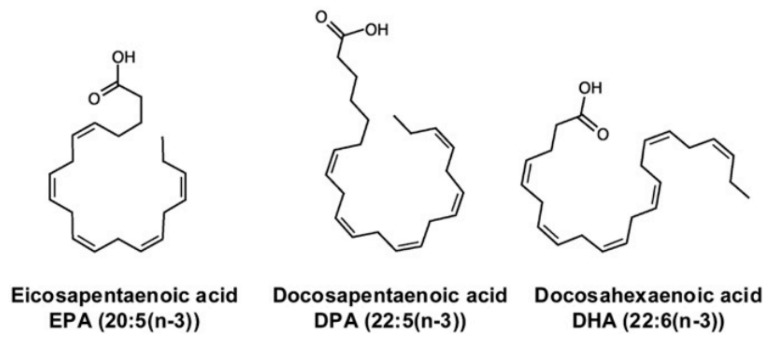
The long-chain n-3 polyunsaturated fatty acids: eicosapentaenoic acid (EPA), docosahexaenoic acid (DHA), and docosapentaenoic acid (DPA) [109].

n-3 polyunsaturated fatty acids (PUFAs) are found in fish and fish oil, and they have several biological properties that might be beneficial in neurodegenerative diseases [102]. The endogenous synthesis of eicosapentaenoic acid, docosahexaenoic acid, and docosapentaenoic acid (Figure 4) within the brain is low compared to their uptake from the plasma, which indicates that their brain levels are maintained via uptake from dietary and/or liver sources via the plasma [103]. Studies conducted in mouse and rat models have shown that n-3 PUFA supplementation can improve neurogenesis, executive functions, and learning abilities, whereas n-3 PUFA deficiency is linked to memory deficit and impaired hippocampal plasticity [104]. Improvements to mitochondrial function in in-vivo and ex-vivo studies using animal models of aging and neurodegenerative diseases have been reported after treatments with PUFAs [105]. The n-3 PUFAs eicosapentaenoic acid and docosahexaenoic acid have anti-inflammatory activities, decrease age-related microglial activation and oxidative stress, and increase the levels of pro-inflammatory cytokines [106,107,108]. Even though the data from clinical studies have been somewhat conflicting, n-3 polyunsaturated fatty acids appear to show neuroprotective properties that are potentially beneficial in the treatment of numerous neurodegenerative and neurological diseases.

## 3. Mechanisms of Neuroprotection

An extensive review by Reglodi et al. described the recent approaches for neuroprotection in PD, with a particular focus on the role of antibiotics, polyphenols, and neuropeptides [70]. The main mechanisms of neuroprotection identified include: (i) oxidative stress, (ii) neuro-inflammation, (iii) protein fibrillation, and (iv) mitochondrial dysfunction.

### 3.1. Oxidative Stress

Numerous studies have reported on the antioxidative properties of polyphenols in neurons, although it is only recently that the focus has turned to their roles in the reduction of intracellular ROS levels [110]. Pro-oxidant toxins (e.g., 6-hydroxyDA [6-OHDA], rotenone, paraquat, 1-methyl-4-phenylpyridinium [MPP+]) have been used in in vitro PD models to clarify these mechanisms. On the other hand, in vivo studies have offered deeper insights into the behavioral improvements conferred by polyphenol treatments, as well as into the improved oxidative status. Recently, interest in the implication of the Keap1/Nrf2/antioxidant responsive element (ARE) axis that underlies the polyphenol antioxidative actions has increased significantly [110]. It has been shown that in human neuroblastoma DAergic SHSY5Y cells treated with 6-OHDA and pretreated with the flavonoids pinocembrin [111] or naringenin [112], a citrus fruit flavanone, and another representative of the natural polyphenols, there was reduced formation of ROS. An increase in the Nrf2 protein levels was induced, which subsequently activated the genes of the ARE pathway. It was also noted that naringenin silenced Nrf2, which resulted in reduced beneficial effects [112]. Furthermore, with the MDA-MB-231-ARE-Luc stable cell line, naringenin activated Nrf2 transcriptional activity, with a dose-dependent increase in ARE-dependent luciferase gene activity. The same study showed that in 6-OHDA–lesioned mice, oral administration of naringenin also activated the Keap1/Nrf2/ARE axis, and oxidative stress was decreased [112]. A recovery of striatal tyrosine hydroxylase was also seen, which is a key enzyme in DA synthesis. Consequently, the concentration of DA and its metabolites were increased, and apomorphine-induced rotations in this mouse model were reduced. Another study showed that oral administration of puerarin to 6-OHDA–lesioned rats reduced apomorphine-induced rotations at the same time as the levels of DA and its metabolites were increased in the substantia nigra [113]. However, this polyphenol treatment also increased the expression of Nrf2 and Keap1.

It has also been shown that polyphenols can increase the expression and/or activity of endogenous antioxidative enzymes. Several studies have shown that the glutathione (GSH), which is one of the most efficient intrinsic antioxidant, functions can be improved by polyphenols. Thus EGCG [114], baicalein [115], and nephrocizin [116] improve GSH levels as well as GSH peroxidase activity after oxidative insult in vitro. Additionally, it has been observed that several polyphenols reduced DAergic degeneration, improve the motor deficit [117,118,119,120,121] and apomorphine-induced or amphetamine-induced turns in rodent models [112,113,122,123]. Furthermore, an aqueous extract from the fern *Selaginella delicatula*, which is a rich source of bioflavonoids, improved GSH levels and increased the activity of GSH S-transferase, which catalyzes the addition of GSH onto potentially harmful xenobiotic substrates for their detoxification [124]. It was also shown that puerarin improved GSH levels in the brains of 6-OHDA–treated rats by causing a simultaneous increase in γ-glutamylcysteine synthetase, which is involved in GSH synthesis [123]. Furthermore, some other antioxidative enzymes were studied after polyphenol treatments, including superoxide dismutase (SOD), which dismutes the superoxide anion in the mitochondria (MnSOD), cytosol (CuZnSOD), catalase, which detoxifies H_2_O_2_,, and AD(P)H:quinone oxidoreductase-1, which prevents the one-electron reduction of quinones and production of radical species. Substantial increases in the activities of these enzymes and dampened motor deficit were seen, particularly in the presence of puerarin [113], curcuminoids [117], and pycnogenol [120] found in an extract of *Pinus maritime* bark containing the proanthocyanidins as main polyphenolic constituents.

It is well known that ROS production and mitochondrial homeostasis are strongly connected. Several studies have investigated the role of oxidative stress and the proteins involved in mitochondrial function (e.g., those of the ‘Silent mating type information regulation 2 homolog 1′ – ‘Peroxisome proliferator/activated receptor gamma coactivator’ 1-alpha [SIRT1/PGC-1α] axis). They have shown the significant increase in the expression of SIRT1 and PGC-1α, with improvements in transcriptional activity after flavanol EGCG administered to MPP+-treated differentiated DAergic PC12 (rat pheochromocytoma) cells [114]. It is well established that 6-OHDA auto-oxidizes in cell-culture medium to yield the cytotoxic by-products H_2_O_2_ and p-quinone, which then promote the production of ROS. Further, 6-OHDA reacts with cysteine residues and forms noxious quinoproteins.

Lin et al. [116] showed that treatment of differentiated DAergic PC12 cells by using nephrocizin (luteolin-7-O-b-d-glucopyranoside) prevented cell death from exposure to H_2_O_2_ or p-quinone. However, nephrocizin had no effects on 6-OHDA auto-oxidation and the formation of H_2_O_2_ in the absence of the cells. It is likely that nephrocizin suppresses the production of intracellular ROS through H_2_O_2_, but not through p-quinone. Interestingly, nephrocizin inhibited the activation of pro-apoptotic substances (e.g., caspase-3, caspase-8), which was mediated primarily through H_2_O_2_ and p-quinone. Additionally, nephrocizin is capable of diffusing through the cell membranes and scavenges the hydroxyl radical directly. Similarly, Meesarapee et al. [125] showed that pretreatment of human neuroblastoma SH-SY5Y cells exposed to 6-OHDA by curcumin effectively reduces cell death by decreasing the levels of quinone-bound proteins.

Polyphenols also modulate the mitogen-activated protein kinase (MAPK) pathways that is involved in cell survival and apoptosis, and might have roles in the protection against oxidative stress. For example, curcumin [125] and naringenin [112] reduced the phosphorylation of MAPKs and the levels of cleaved caspase-3 in human neuroblastoma SH-SY5Y cells exposed to 6-OHDA. Curcumin and naringenin also prevented intracellular Ca^2+^ increases, ROS production, and decreased apoptosis through mitigation of the expression and activity of pro-apoptotic factors [126]. Similarly, rutin (quercetin-3-O-rutinoside) repressed phosphorylation of c-Jun N-terminal kinase (JNK) and p38, while the administration of inhibitors of either JNK or p38 mimicked these neuroprotective actions of rutin and potentiated its effect in co-treatments. In another study on primary astrocytes treated with MPP+, the flavone baicalein inhibited phosphorylation of JNK and extracellular signal-regulated kinase (ERK) [127]. Finally, the kinase Akt appears to be another important enzyme involved in the neuroprotection modulated by the polyphenols, as Akt phosphorylation was increased by puerarin administration in mice treated with the neurotoxin 1-methyl-4-phenyl-1,2,3,6-tetrahydropyrimidine (MPTP) [121]. At the same time ROS were reduced, GSH activity was increased, and the motor deficits were improved.

### 3.2. Neuroinflammation

To investigate the anti-inflammatory potential of polyphenols as therapeutic molecules and to explore the immunomodulatory dysfunction that accompanies PD pathophysiology, various in-vitro models of neuroinflammation have been established. Most of these involve glial cells challenged with lipopolysaccharide, a component of the outer membranes of Gram-negative bacteria that activates toll-like receptor 4 (TLR4) to elicit a robust inflammatory response in a wide variety of immune effector cells [128,129]. In these PD models, rotenone, MPP+, and 6-OHDA can also cause neuroinflammatory transformations to glial cells. Gliosis and cytokine dysregulation, which can be seen for brains of patients with PD, are often seen in the rodent models of this disease. Some studies have indicate that various polyphenols can decrease the expression or transcription of pro-inflammatory cytokines (e.g., interleukin-1 beta [IL-1β], TNF-α, IL-6) [115,120,130,131]. For instance, theaflavin treatment diminished the expression of the anti-inflammatory cytokines IL-4 and IL-10, which increased as a compensatory mechanism in mice treated with MPTP or probenecid; this suggests a general effect of dampening of the inflammatory response. It is also possible that the role of the polyphenols in the modulation of the ‘Suppressor of cytokine signaling 1′ (*SOCS1*) gene underlies their anti-inflammatory potential. As such, the protein that is encoded by this gene functions downstream of the cytokine receptors, and furthermore takes part in a negative feedback loop to attenuate cytokine signaling.

Lofrumento et al. showed that orally administered resveratrol reduced glial cell activation, rescued DAergic neurons, and decreased the production of IL-6, IL-1β, and TNF-α (as well as their receptors) in the substantia nigra of MPTP-treated mice [131]. Interestingly, resveratrol up-regulated the transcription and expression of *SOCS1* in the striatum and substantia nigra. This might explain the controlled release of proinflammatory cytokines. As the CNS has both glial cells and neurons, it is important to study their cross-talk in order to elucidate the underlying mechanisms of neuroinflammation. It is important for so called insert co-culture systems to be used here without cell-to-cell contact, as these systems allow the identification of the cell culture that generates the toxic effects, as well as the one that is being affected. As such, when administered to lipopolysaccharide-activated or MPP+-activated N9 microglial cells (murine cell line) that were co-cultured on inserts with differentiated DAergic PC12 cells, resveratrol and quercetin prevented pro-inflammatory cytokine expression and transcription, and also neuron apoptosis [132,133]. Furthermore, in a similar system of rotenone-treated primary mesencephalic neurons with primary microglial cells, resveratrol improved cell survival [134]. Importantly, these beneficial effects were not observed when the neurons were cultivated without the microglial cells, which suggested a neuroprotective effect that is facilitated by the glial cells.

As well as the cytokines, other important pro-inflammatory systems include iNOS and the prostaglandin-synthesizing enzyme COX-2, as well as markers of astrogliosis or microgliosis, such as integrin alpha M (CD11b), glial fibrillary acidic protein, and ionized calcium binding adaptor molecule 1 (Iba1). Several polyphenols (e.g., theaflavin [130], baicalein [127], resveratrol [131], puerarin [113], pycnogenol [120]), reduced levels of these proteins or their mRNAs and in parallel induced DAergic neuroprotection and behavioral improvements. Furthermore, quercetin decreased the activity of NADPH diaphorase in neurons, which catalyzed the production of the inflammatory mediator NO in 6-OHDA–treated rats in a similar way to iNOS [133]. Another study of various rotenone-challenged cell cultures implicated myeloperoxidase (MPO) that produces tyrosyl radical and hypochlorous acid during the microglial cell respiratory burst, which supported a multidimensional anti-inflammatory role for resveratrol [134]. It was shown that the tyrosyl radical and hypochlorous acid are cytotoxic to pathogens and to the cells that produce them, thus linking the neuroinflammation with oxidative stress. The expression of MPO and the levels of nitrite, a NO metabolite was reduced when resveratrol was administered to rotenone-treated BV2 cells (murine microglial cell line) and to primary microglial cells. It was shown that MPO has a role as a positive-feedback element and it can increase its own expression and activity in microglial cells and astrocytes. On the other hand, it was observed that resveratrol can moderate MPO expression and activity in primary microglial cells and astrocytes, which was not seen for other anti-inflammatory agents tested (e.g., ethyl pyruvate, 15-deoxy-D-12,14-prostaglandin J2). Resveratrol also significantly attenuated the rotenone-induced production of nitrite and transcriptional up-regulation of IL-1β, COX-2, TNF-α, and iNOS in MPO-deficient primary glial cells. Finally, this study showed that resveratrol also diminished the expression of MPO and the production of ROS. These anti-inflammatory roles of resveratrol have been intensively studied, and they have been reviewed in greater detail elsewhere [135].

The milk thistle extract silymarin is a very ‘popular’ polyphenol mixture, which is composed of various flavonolignans, in terms of its anti-inflammatory properties. In 6-OHDA–challenged rats, intraperitoneal injections of silymarin provided improvements to their bar catalepsy scores [136] and motor coordination [137]. Similar to resveratrol, silymarin also decreased striatal MPO activity, and decreased the levels of the pro-inflammatory cytokines in the cerebrospinal fluid [136,137]. Anti-inflammatory properties of silibinin, the main flavonolignan of silymarin, was also investigated. When administered to MPP+-treated mice, silibinin was shown to promote the recovery of tyrosine hydroxylase levels and to decrease the expression of both iNOS and the pro-inflammatory cytokines in the substantia nigra [115]. It can thus be concluded that silibinin is very likely at least partially responsible for the potent anti-inflammatory actions of silymarin.

Anti-inflammatory actions are also one of the known effects of the estrogen-like polyphenols, or phytoestrogens. Three soy phytoestrogens daidzein and genistein (isoflavones), and the coumarin coumestrol were investigated in terms of their anti-inflammatory properties in comparison with 17β-estradiol, with lipopolysaccharide-activated HAPI cells (rat microglial cell line) [138]. Here, it was found that all three of these phytoestrogens reduced the transcription and expression of iNOS. They also decreased the production of NO, through an antioxidation-independent mechanism. These phytoestrogen treatments also mitigated the expression of the chemokine ‘Monocyte chemoattractant protein-1′ (MCP-1) and the pro-inflammatory cytokine IL-6. These data for the polyphenolic phytoestrogens were similar to those obtained with 17β-estradiol, which indicated a possible binding effect of estrogen receptors. These data can also explain the independent mechanism of ROS scavenging for neuroprotection at low concentrations (micromolar ones).

However, the studies investigated the anti-inflammatory effects of the polyphenols with the aim to define the cellular mechanisms are scarce. Lee et al. [127] applied baicalein to MPP+ treated primary astrocytes and reported that it inhibited nuclear translocation of the pro-inflammatory power player ‘Nuclear factor kappa-light-chain-enhancer of activated B cells’ (NF-κB). Indeed, reduction of the nuclear localization of this transcription factor decreased the expression of the downstream target COX-2, which explains the role of the NF-κB pathway in the anti-inflammatory actions of baicalein. NF-κB transcriptional activity also increases the expression of most pro-inflammatory cytokines and enzymes. Consequently, the *Pinus maritime* bark extract pycnogenol decreased the expression of NF-κB as well as its downstream targets [120]. Finally, further studies are needed to define the signaling mechanisms through which the polyphenols act to offset neuroinflammation.

### 3.3. Protein Fibrillation

Epigallocatechin-3-galate, the most abundant of the flavanols in green tea, is one of the most interesting polyphenols involving PD and AD, due to its effect on destabilization of fibrils [139], and type 2 diabetes [140]. EGCG prevented the formation of toxic aggregation of amyloidogenic α-synuclein in vitro [22,139,141,142]. It is believed that EGCG promotes ‘off-pathway’ oligomer formation, which is nontoxic in mammalian cell lines, such as human embryonic kidney-293 (HEK-293) and rat PC12 cells [139]. Additionally, EGCG protected the cell membrane from being destabilized by preformed fibrils [141]. Similar effects on α-synuclein oligomer formation were seen for 14 naturally occurring polyphenolic compounds and for a black tea extract. All of these studied polyphenols inhibited α-synuclein aggregation in a dose-dependent manner. Additionally, they promoted disaggregation of pre-formed oligomers [143] and reduced the levels of α-synuclein oligomers in the striatum and hippocampus. The mechanisms of EGCG action remain unknown, however, although it is believed that EGCG has protective effects through the production of off-pathway ‘compact’ oligomers and by facilitating the conversion of ‘active’ oligomers into amyloid fibrils [144].

To test the EGCG fibril-destabilizing effects in vivo, mice underwent oral administration of EGCG followed by MPTP treatment; here, EGCG prevented α-synuclein accumulation [68]. Further, a mixture of polyphenols from green tea that included various catechins and in particularly EGCG was shown to reduce motor deficits in MPTP-lesioned cynomolgus monkeys, through increased numbers of substantia nigra tyrosine-hydroxylase-positive neurons and increased DA levels in the striatum [145]. Strong neuroprotection by green tea was also observed in an Alzheimer’s-like rat model, with reduction of the memory deficits and the levels of ROS and thiobarbituric-acid-reactive substances in the hippocampus. Based on these data, green tea supplements can be used to reduce the effects of AD due to their high EGCG concentration [19].

In parallel, it was shown that polyphenols from tea lowered the levels of oligomers while enhancing the cell viability in a DAergic substantia nigra/neuroblastoma hybrid cell line known as MES23.5 cells, when they were treated with the combination of MPP+ and α-synuclein monomers or oligomers. Other polyphenols have also shown fibril-destabilizing properties through inhibition of initiation and/or propagation of α-synuclein fibrils in vitro, including quercetin [146,147], curcumin [147,148,149], and its metabolites [150], chlorogenic acid [151], and resveratrol [147]. Interestingly, the oxidized form of quercetin was more efficient at destabilization of human wild-type α-synuclein formation of fibrils and their growth [146]. Although quercetin and its oxidized species disaggregated preformed fibrils, it is likely that oxidized quercetin is more potent because of its higher hydrophilicity and polarity.

*Drosophila melanogaster* that expresses wild-type human α-synuclein is a good model for investigating the synucleinopathic aspect of PD and for studying the promissing neuroprotective strategies [135]. Curcumin and EGCG added to the *Drosophila*’s diet promoted the increased life span, recovered the loss of climbing ability, reduced protein carbonyl content as well as lipid peroxidation, and mitigated the death of brain cells [152,153,154,155]. Another strategy that should be considered in the prevention of protein fibrillation is the removal of oligomers and aggregates that is regulated by autophagic pathways that are impaired in PD [156]. The treatment of differentiated DAergic cells (PC12) with the inhibitors of the proteasoms (e.g., MG132 and MG115) resulted in extensive mitochondrial damage and cell death, including apoptosis. The flavone baicalein inhibited these effects following proteasome inhibition [115]. Furthermore, it was shown that resveratrol protected human neuroblastoma cells (SH-SY5Y) against death when challenged with rotenone [157]. A role for autophagy was in this process suggested when bafilomycin A1, an autophagosome–lysosome fusion inhibitor, was administrated and it abolished this process. Additionally, resveratrol-mediated autophagy and neuroprotection can be abolished with pharmacological inhibition of an enzyme, namely heme oxygenase-1. Similarly, it was shown that puerarin increased the expression of membrane protein 2a (LAMP2a), which is lysosome-associated and works as a lysosomal membrane receptor that is involved in regulation of the removal of cytosolic proteins by means of chaperone-mediated autophagy [121]. Here, the levels of LAMP2a present at the lysosomal membrane were directly associated with the proteolytic pathway. Even if there was no direct evidence of removal of α-synuclein oligomers, it was found that quercetin inhibited Aβ-induced paralysis in *Caenorhabditis elegans* through activation of protein degradation pathways [158]. Evidently, a role for modulation of protein misfolding and clearance underlies the neuroprotective actions of some polyphenols [70,159].

Naringenin was shown that for MPTP-induced α-synuclein in a mouse model, naringenin significantly down-regulated α-synuclein and up-regulated DA transporter and tyrosine hydroxylase protein expression [160]. Additionally, it down-regulated TNF-α and IL-1β mRNA expression, and improved levels of superoxide dismutase. Naringenin lowered also levels of GSH compared to the mice with vehicle-treated PD. Up-regulation of tyrosine hydroxylase with naringenin treatment corresponded to increased homovanillic acid and DA (3,4-dihydroxyphenylacetic acid) turnover, and motor functions. It is likely that naringenin can possibly counteract DAergic degeneration, induced by MPTP, through regulation of neuroinflammation, α-synuclein fibrillation, and oxidative stress [144].

Oleuropein aglycone is another example of the natural polyphenols, and it is the main olive oil polyphenol. It has been the subject of many studies due to its anti-amyloidogenic activity in vitro. It was shown that oleuropein aglycone stabilized α-synuclein monomers, and prevented the multiplication of on-pathway oligomers, which favored the growth of very stable and harmless aggregates that did not evolve into cytotoxic amyloids. Oleuropein aglycone prevented α-synuclein aggregates from binding to cell membrane components, which resulted in prevention of oxidative damage to cells and reduction of cytotoxicity [47,161].

### 3.4. Mitochondrial Dysfunction

In PD, pathophysiological dysfunction of the mitochondria has been seen in models using toxins that target the electron transport chain. Initially, the loss of mitochondrial transmembrane potential was observed, followed by apoptosis of DAergic neurons. Furthermore, different polyphenols have been investigated in vitro in terms of the possibility to recover the mitochondrial transmembrane potential after oxidative damage [115,162]. Here, in-vivo studies had previously shown reduced DAergic degeneration and improvement of motor deficits [124,163]. The rotenone-challenged mice were administered water extract of *S. delicatula*, which improved the activities of complexes I and II as well as citrate synthase, and the amounts of expressed mitochondrial ATPases [124]. A similar benefit was shown for quercetin in 6-OHDA–treated rats, which resulted in improved activity of complex I in the substantia nigra through reduction of DNA fragmentation that suggested powerful anti-apoptotic actions [122]. When silibinin was orally administered to rats that were pre-treated with MPP+, the levels of complexes IV and V (ATP synthases) were improved [163]. At the same time, the decrease in complex I activity, induced by MPP+, was followed by an increase in the levels of complex II in transport chain.

To better understand the neurotoxic and neuroprotective effects of toxins and polyphenols, studies on isolated brain mitochondria are desirable. As such, when isolated rat brain mitochondria treated with 6-OHDA were administered baicalein, the ROS production was reduced while the lipid peroxidation followed by the presence of FeSO_4_-cysteine scenario was prevented [162]. In another study performed on rat cortex mitochondria in the presence of rotenone ex vivo, quercetin scavenged the hydroxyl radical. These studies suggest an immediate action of quercetin in ROS depletion [122].

The genes involved in the most common forms of PD are often associated with mitochondrial homeostasis. It has been shown that DJ-1, Leucine-rich repeat kinase (LRRK)2, PTEN-induced kinase (PINK)1 and Parkin act in the dynamics of fission/fusion, lysosomal-autophagic degradation, mitophagy, and mitochondrial ROS sensing [164]. The expression of DJ-1 in 6-OHDA–treated SH-SY5Y human neuroblastoma cells was increased by the flavone baicalein [162]. In the presence of EGCG, the neurodegeneration was reversed by DAergic and substantially improved the climbing activity of G2019 LRRK2-transgenic or Parkin-null *Drosophila* [165]. However, the protective effects of EGCG were diminished, when was genetically inactivated. Resveratrol is another polyphenol where its influence on AMP-activated protein kinase has been widely investigated. Indeed, this polyphenol improved mitochondrial homeostasis in primary skin fibroblast cultures, obtained from two patients suffering from early onset PD followed by *Park2* mutations [166]. The same polyphenol increased the mitochondrial function through increased activity of complex I, ATP production, consumption of oxygen, and a concurrent decrease of ROS production and lactate content. Additionally, it has been shown that resveratrol is involved in many other processes, such as an increased NAD+/NADH ratio, and activation of AMPK and SIRT1. It was also shown that resveratrol modulated PGC-1α transcriptional activity, as seen by increased mRNA expression of many downstream genes. In skin fibroblast cultures, it also increased the macro-autophagic flux through a microtubule-associated protein light-chain-3–independent pathway, and consequently through α-synuclein clearance.

## 4. Therapeutic Considerations

In preclinical PD models, polyphenols show strong neuroprotective properties. Due to their proven valuable effects in terms of neuroinflammation, oxidative stress, mitochondrial dysfunction, and protein misfolding for patients with PD, it is worth investigating other polyphenols further in addition to green tea [69]. This neuroprotection is likely to be due to the polyphenol actions at the molecular level, through MPO, heme oxygenase-1, α-synuclein fibrillation, and various pathways, such as the SIRT1/AMPK, MAPK, and Keap1/Nrf2/ARE axes. As described in this review, the most studied polyphenols are quercetin, resveratrol, curcumin, and EGCG. Moreover, silymarin extract, rich source of baicalein, and silibinin have gained lots of interest only recently.

Although the polyphenols have many positive effects that have been seen in model systems, there remain a few issues in the pre-clinical design, where they have appeared to be less predictive for human applications. Indeed, in terms of the importance of polyphenols’ direct antioxidant activity in physiological conditions, the following need further consideration: (i) transfer of H-atoms must occur faster than reactions involved in the free-radical production cascades [167]; (ii) the levels of polyphenol in tissues or plasmar at any given time, which will be submicromolar [168]; and (iii) that their concentrations are lower than the ones from endogenous antioxidants, for instance ascorbate (30-100 mM) and urate (140–200 mM). Indeed, the polyphenols do not contribute more than 2% to the total plasma antioxidant activity, and this is not likely to be relevant in a (patho)physiological context [169]. Based on this, it is more likely that the biological effects of polyphenols happen at their nanomolar concentrations, involving indirect mechanisms, such as enhancing the Keap1/Nrf2/ARE pathway. Such indirect mechanisms of the polyphenol actions might better explain their antioxidative properties. Furthermore, the main consideration regarding the use of polyphenols in the treatment of neurodegenerative diseases are both, their bioavailability and the ability to cross BBB.

Most drugs are administered orally, and thus their pharmacological effects are dependent on their oral bioavailability [56]. The bioavailability and bioefficacy of drugs depend on: (i) their interactions with the food matrix; (ii) their physicochemical properties; and (iii) the physiological conditions of gastrointestinal tract. The great interest in phytochemicals is due to their potential use as ‘alternative’ drugs and for the beneficial effects that arise from their nutritional intake. The pharmacokinetics profiles of polyphenols after oral administration are thus an important issue [170].

The bioavailability of polyphenols is very important with respect to their therapeutic administration. The majority of polyphenols are highly glucuronidated or sulfated in human plasma. EGCG is one of the polyphenols that is known not to be transformed in this way, and hence is abundantly available in human plasma in its free form (77%–90%) [171]. After digestion in the stomach, several polyphenols do not change their structure, whereas the others are attacked by the digestive enzymes in the duodenum, and are transformed into galloyl or methylated derivatives that show decreased absorption [17]. For example, after single-dose administration of >1000 mg EGCG in humans, the pharmacokinetics profile revealed about 1 mM of maximal plasma levels [171]. Further, it is worth noting that the bioavailability of EGCG depends not only on the consumption dose, but also on the frequency of its administration [172]. Another example is stilbenoid resveratrol, which is in the intestine and liver easily metabolized into its sulfate (*trans*-resveratrol-3-sulfate) as well as into its glucoronide (*trans*-resveratrol-3-O-glucuronide) conjugates [53]. Nevertheless, some recent studies have shown that these metabolites might also provide significant benefits [150,173]. It should also be noted that resveratrol sulfates and other sulfated polyphenols can be transformed back to their original states by using human-expressed sulfatases [174].

Finally, most polyphenols that are orally administered using their natural sources do not represent any notable safety hazard. However, some studies have determined irritation of gastrointestinal tract. On the other hand, some caution is required when consuming these substances in higher quantities in the form of food supplements [18,22]. Potential toxicities of polyphenols are likely to arise from: (i) pro-oxidant effects connected to their radical-scavenging properties; (ii) excessive biotransformation in the liver; and (3) increased acute stress responses. The pro-oxidant activities of polyphenols can be attributed to the catechol’s structure; catechols can generate superoxide anions, which can lead to formation of the quinone counterparts [175].

The most encouraging results regarding improved bioavailability of these polyphenols to the CNS will be novel approaches in the development of their delivery systems through encapsulation technologies [176]. Indeed, the bioavailability of polyphenols has been improving consistently through pharmacological advances, such as with the use of nanoparticles, lipid nanocapsules, nanocomposites, exosomes, and emulsified formulations [177]. It was shown that the intranasal administration of polyphenols could enhance their bioavailability, similarly to the promising results obtained with other CNS-directed drugs, for instance insulin for AD or apomorphine for PD [178]. Indeed, it was shown that curcumin was better incorporated into the brain when administered intranasally compared to the oral administration [179]. However, there are some risk factors that need further assessment, such as nasal irritation, which might be the major drawback [178].

## 5. Conclusions

Many dietary compounds, and particularly polyphenols, are available in the form of dietary supplements for ‘treatment’ of different diseases, namely diabetes, and related metabolic disorders as well as widely spread neurodegenerative diseases. However, due to wage regulation, their effectiveness is usually not supported by appropriate scientific evidence. Even if several studies, performed on in-vitro and in-vivo models, have reported positive effects of polyphenol supplements, the results remain conflicting. Recently, a comprehensive review has been published by Poti et al. [180] who critically considered and meta-analyzed randomize controlled clinical trials involving subjects taking polyphenol – based supplements. They found out that some polyphenols might improve specific markers of cognitive status and cardiovascular risk. Therefore, further studies have to be performed not only to confirm their positive effects, but also more seriously address the safety issues. This can be obtained by conducting several well-designed, placebo-controlled, double-blind clinical studies. Only then can the safe use of polyphenols supplements be proposed for treatment and/or prevention of neurodegenerative diseases.
